# Injury rates following conducted electrical weapons and other less-lethal force modalities in real-life police settings: a comparative literature review

**DOI:** 10.1007/s12024-025-01020-9

**Published:** 2025-05-15

**Authors:** Mark Nielsen, Julie Munkholm, Jytte Banner, Carl Johan Wingren

**Affiliations:** Department of Forensic Medicine, Section of Forensic Pathology, University of Copenhagen, Frederik’s V’s Vej 11, Copenhagen, 2100 Denmark

**Keywords:** Clinical medicine, Forensic medicine, Conducted electrical weapons, Use of force, Police

## Abstract

The aim of this study was to rank the rate and risk ratio of subject injury following exposure to less-lethal force modalities in real-life police interventions. The purpose was to observe whether the use of conductive electrical weapons (CEW) qualifies as a low-risk modality of force, with a similar risk of adverse health outcomes as exposure to other less-lethal modalities, such as physical force, baton, oleoresin capsicum (OC) spray, and canine. A literature search was conducted using the scientific databases PubMed, Embase, and Web of Science. We rated the included articles based on quality and calculated the weighted means of injury rates. We found 19 articles reporting the rate or risk ratio of injury following exposure to common less-lethal force modalities in real-life settings. OC spray appeared to have the lowest rate of subject injury. The outcome for subject injury following CEW was ambiguous but seemed to be lower in comparison to baton and canine. However, the findings should be interpreted with caution due to methodological limitations. The identified studies were heterogeneous in design, which limits the conclusions drawn. In general, there was an indication that the risk of injury is lowest when OC spray is used. The rate of subject injury following the use of CEW is lower compared to baton and canine. Further research using a systematic approach to the registration of injuries is warranted.

**Supplementary Information:**

The online version contains supplementary material available at 10.1007/s12024-025-01020-9.

## Introduction

Less-lethal weapons are regarded as relatively safe alternatives to lethal weapons with the intention to subdue subjects [1]. These modalities include conducted electrical weapons (CEW), chemical irritants such as oleoresin capsicum (OC) spray and tear gas, and canine and kinetic impact projectiles (e.g., rubber bullets, bean bags) [2]. On the use-of-force (UOF) continuum, these modalities are ranked between lethal force and physical force and equal to the use of baton [3].

Nonetheless, all less-lethal force methods are associated with the risk of injury. The risk for injuries associated with each type of force could condition the surrounding legal framework and be of importance for tactical guidelines. This knowledge could also aid in determining whether exposure to a force method should routinely include a medical examination. Determining risks for injury associated with each modality is no easy task, since the risks are determined not only by the characteristics of the force but also by their tactical use, the aggressor in the situation at hand, the preexisting health of the persons exposed, and the fact that several approaches can be used in the same situation. To be able to correctly assess the risk of adverse health outcomes following exposure to UOF, it is important that exposed persons are systematically examined for lesions and other adverse health outcomes, and that the effects of each modality are ascertained.

CEWs fire probes that attach to the body and pass pulsed electricity into the exposed person through wires. Electricity elicits involuntary muscle contractions, rendering the subject unable to control their body and allowing law enforcement to control the situation. Some models of CEW can also be used in drive stun mode: CEW is pressed against the subject’s body, releasing electricity into the body. In contrast to the probe mode, no efficient muscle contractions can be elicited in the drive stun mode; rather, the effect is the sensation of pain [4].

In probe mode, adverse health effects are related to (i) the electricity, such as cardiac arrythmias [5, 6] and rhabdomyolysis [7, 8], (ii) the impact of the probes (e.g., fractures of minor bones [9], pneumothorax [10] and eye injuries [11, 12]), and (iii) secondary injuries (e.g., head traumas following falls due to the loss of voluntary muscle control [13]). Adverse health effects observed in exposed persons are often derived from case reports, but such effects are low in the evidence hierarchy [14]. Consequently, the findings should be interpreted with care. In particular, this is true of adverse effects on the heart, which have been subject to debate [15, 16].

Experimental research on healthy subjects has further increased knowledge of adverse health effects following exposure to CEW [17, 18]. In such experimental settings, there have been no observations of significant changes in blood levels in terms of troponin, creatine kinase, cortisol, catecholamines, lactate, and alpha-amylase, as well as heart rate or blood pressure [17, 18]. In terms of ventilation, the findings in one study were mixed, indicating that the effect on ventilation might depend on CEW exposure length [18]. In general, CEW exposure to healthy adults does not seem to imply a significant health risk [17]. This conclusion aligns with the most recent systematic review of adverse health effects following CEW [19]. However, almost all included studies were performed in safe clinical settings on healthy volunteers. Furthermore, several of the studies were funded by CEW manufacturers. Thus, the findings in these controlled studies on safety might not allow for extrapolation to real-life settings in which subjects exposed to CEW could have preexisting health issues or be under the influence of pharmaceuticals or illegal substances; hence, further research is needed.

Using the baton and physical force can cause blunt force trauma that might result in hematoma and fractures, and the use of various restraining techniques carries the risk of asphyxia by restricting breathing or blood flow [20]. OC spray is a product from pepper plants that can cause immediate acute burning pain, swelling of the skin and eyes, and inflammation of mucus membranes, rendering breathing more labored. Moreover, systemic symptoms, including loss of body motor control, hyperventilation, tachycardia, and pulmonary edema, have been reported in cases where OC spray was used [21]. The use of canines as a modality of force ranges from no-contact intimidation techniques (such as barking) to “bite-and-hold” strategies. This method carries risks for soft tissue, nerve, and vascular injuries of various severities [22].

It is essential to study health outcomes following UOF in real-life settings, as the risks of injury associated with each modality are likely conditioned by tactical decisions, the level of agitation in the situation, preexisting health conditions, and possibly any influence of drugs on the exposed person. Thus, the aim of this study was to rank the rate of subject injury following exposure to less-lethal force modalities in real-life police interventions. The purpose is to observe whether the use of CEW by law enforcement qualifies as a low-risk modality of force, with similar risks and severities of adverse health outcomes following exposure to physical force, baton, canine, and OC spray.

## Materials and methods

A literature search was conducted on January 8, 2025, using the scientific databases PubMed, Embase, and Web of Science. We combined different search terms for less-lethal force modalities– physical force, baton, canine, OC spray, and CEW– with injuries and police UOF (see Supplement 1 for search strings).

Inclusion and exclusion criteria were set up using the PICO framework (Table 1). Articles on the epidemiological observations of subjects exposed to less-lethal UOF modalities by law enforcement in real-life settings were included. Notably, we included specifically physical force, baton, canine, OC spray, and CEW. Articles on other less-lethal modalities, such as sponge grenade bullets and bean bags, were excluded. Studies lacking information on which modality had caused the injury and injury inflicted by unintentional UOF (e.g., injuries inflicted due to “weapon confusion”) were also excluded. The outcome was the rate or risk ratio of sustained injury following subject exposure to UOF. Letters to the editor, reviews, narratives, research letters, commentaries, editorials, and case reports, as well as studies published before 2000, were excluded. In addition, the articles had to be written in English or German.

**Table 1:** Inclusion and exclusion criteria

	Inclusion	Exclusion
Population	• Subjects exposed to police/law enforcement UOF	• Officers exposed to violence by subjects • Subjects exposed to violence by other subjects
Intervention/exposure	• Less-lethal police UOF modalities: physical force, CEW, baton, canine, OC spray	• Other types of less-lethal police UOF modalities, such as sponge grenade bullets, attenuating energy projectiles (AEP), kinetic impact weapons such as rubber bullets, plastic bullets, bean bag, shot pellets • Absence of information on what type of modality had caused injury/injuries • Unintentional UOF leading to injury
Comparator/context	• Real-life setting of police interventions	• Other types of less-lethal police UOF as listed under intervention
Outcome	• Rate or risk ratio of sustained injury to a subject caused by a less-lethal modality of police UOF	
Study characteristics	• Observational, epidemiological studies	• Letters to editor, narrative reports, commentaries, editorials, case reports, experimental studies, abstract only, research letter
Other	• Language: English, German • Years: 2000–2025 (Jan)	• All other languages than English and German

**Fig. 1:**
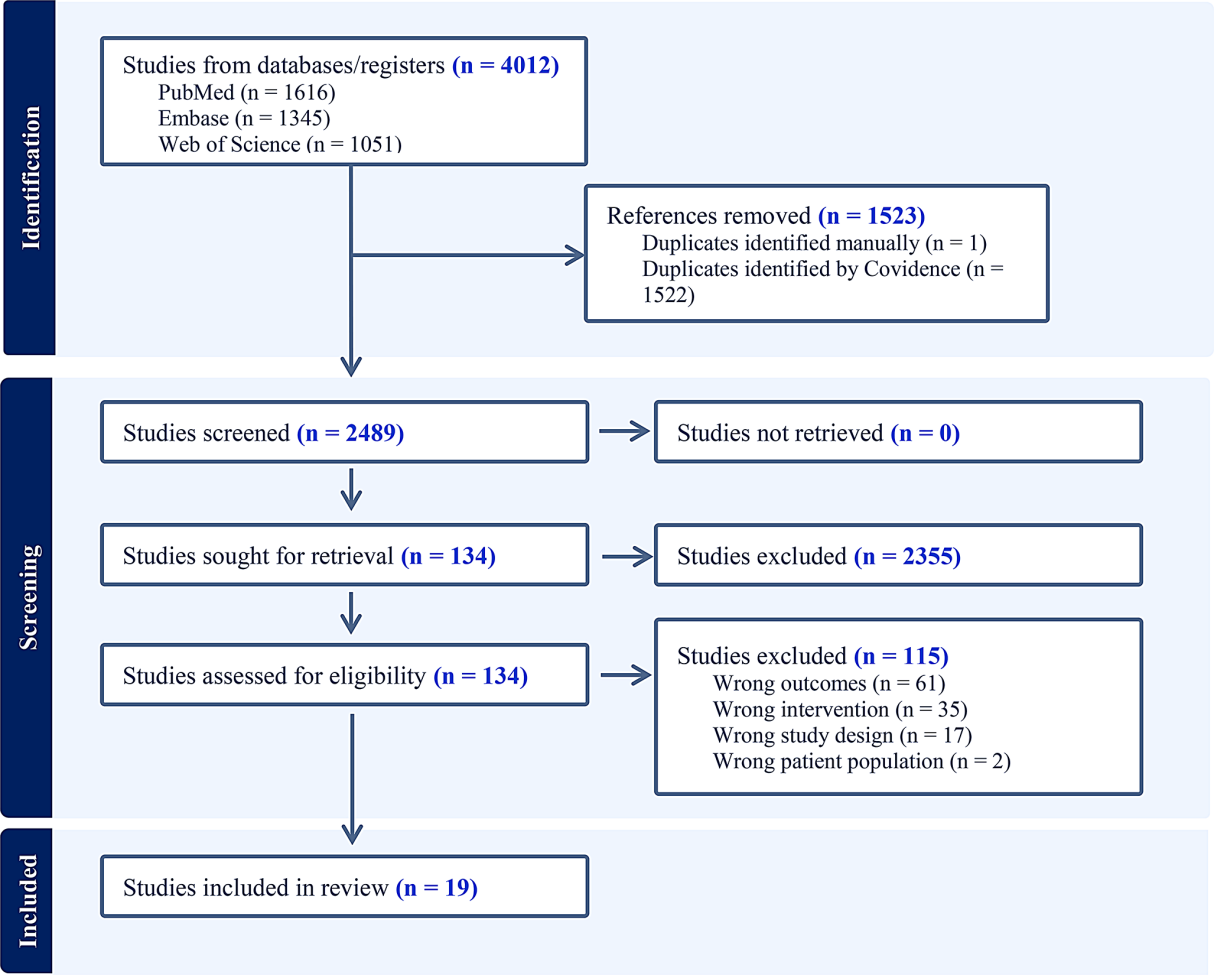
Prisma flow diagram

In total, 2,489 articles were initially screened on title and abstract, of which 134 articles were eligible for full text review, and 19 of those articles met the inclusion criteria (Fig. 1).

Quality assessment of the 19 articles was performed by applying a modified study quality assessment tool [23], focusing on three qualities: (i) population, (ii) time interval, and (iii) identification of injuries. These were to be clearly defined and without signs of bias. Based on this assessment, the articles scored A (high quality) or B (low quality). High-quality articles included all cases subject to police interventions in a defined geographical area, during a specified time, and with transparent reporting on how injuries were registered. The article *Injuries associated with police use of force* by Bozeman et al. [24] is an example of an article assessed as high quality (quality A). It includes all cases exposed to use of force in three mid-sized police agencies during 2011–2012, and injuries were observed by police officers and registered in a use of force database. Authors CJW and MN performed the assessment, and any disagreements were resolved by discussion.

With regard to the rate of injury following less-lethal use of force modalities, we calculated the mean injury rates and the weighted mean injury rates of all included articles, as well as the high-quality articles. The latter was performed to account for differences in incident sizes. Each injury rate was weighted by the corresponding number of incidents, ensuring that articles with higher incident number had a greater influence on the overall estimate. The weighted sum of the injury rates was divided by the total number of incidents. This approach provides a more representative measure of the overall trend by preventing articles with lower incident numbers from disproportionately affecting the result. Finally, the corresponding standard deviations were calculated with following formulas:

1. Mean injury rate = $\:{sd}_{sample\:mean\:}=\sqrt{\frac{\varSigma\:({x}_{i}-\overline{x}{)}^{2}}{n-1}}$
2. Weighted mean injury rate = $\:{sd}_{weighted\:mean\:}=\sqrt{\frac{\sum\:{{w}_{i}\text{*}({x}_{i}-\:{\stackrel{̄}{\text{x}}}_{w})}^{2}}{\sum\:{w}_{i}}}$

$$\:{\text{s}\text{d}}_{\:}=\text{s}\text{t}\text{a}\text{n}\text{d}\text{a}\text{r}\text{d}\:\text{d}\text{e}\text{v}\text{i}\text{a}\text{t}\text{i}\text{o}\text{n}$$, $$\:{\text{x}}_{\text{i}}=\:$$$$\:\stackrel{̄}{\text{x}}$$ = mean injury rate, n = number of references, $$\:{\text{w}}_{\text{i}}=\text{incident number}$$, $$\:{\stackrel{̄}{\text{x}}}_{\text{w}}=\text{w}\text{e}\text{i}\text{g}\text{h}\text{t}\text{e}\text{d}\:\text{m}\text{e}\text{a}\text{n}\:\text{i}\text{n}\text{j}\text{u}\text{r}\text{y}\:\text{r}\text{a}\text{t}\text{e}$$, $$\:\sum\:{\text{w}}_{\text{i}}$$ = total sum of incident numbers.

## Results

We identified 19 peer-reviewed articles reporting on subject injury rates and risk ratios after exposure to less-lethal force modalities during police interventions in real-life settings. Most articles retrieved data on UOF incidents from one source, as the article by Maguire that retrieved information from one source reporting on 2119 UOF incidents [25]. In contrast, three articles retrieved data from more than one source, such as the article by Jenkinson et al. retrieving information from 6 different sources [26]. Hence, information was based on information from a total of 26 sources (Table 2). In terms of context, most of the sources were from the US (*n* = 22), followed by the UK (*n* = 3) and Canada (*n* = 1). Subjects exposed to police less-lethal UOF were predominantly men (75.8-94%) (Table 2). Data spanned from 1998 to 2020; however, four articles did not report on year of observation [26–29]. Regarding data sources, 22 sources concerned CEW, 15 concerned physical force, 14 concerned OC spray, 10 concerned canine, and 9 concerned baton (Table 2). Four articles contained information on models of CEW: “pre-M26-weaponry”, M26 and X26 [24, 26, 30, 31] (Table 2). A few articles used composite grouping of force modalities: *Less-lethal weapons* (OC spray, pepper ball launchers, CEWs, rifles and shotguns using less-lethal shells, and riot shields), *other physical methods* (various forms of physical force, impact weapons such as batons, canines, and the forcible stop of a fleeing vehicle), and *impact weapons* (batons, flashlights, tear gas, and rubber bullets) [32, 33].

**Table 2:** Descriptive statistics, including author(s), years of data, number of data, civilian male sex, types of use of force modality and quality assessment score

Ref	Author(s), years of data	Source	Data #	Male sex	Use of force modalities	Quality assessment
27	Ariel et al., UK -		5981 UOF incidents	-	4	B
38	Bozeman et al., US 2005–2008		1201 subjects	94%	4(X26/M26)	A
24	Bozeman et al., US 2011–2012		893 UOF incidents 914 subjects 1399 force utilizations	89%	1,2,3,4,5	A
32	Castillo et al., US 2009		1174 UOF incidents	75.8%	6,7	A
34	Chang et al., US 2003–2011	1	715,118 nonfatal injuries	84%	1	B
	2003–2011	2	3958 patients	86.5%	1	
35	Haileyesus et al., US 2005–2008		6002 nonfatal injuries	90.1%	1,2,3,4,5	B
39	Hickman et al., US 2014–2018		10,564 subject-incidents	82.6%	1,2,3,4,5	B
26	Jenkinson et al., US US, 1999–2002	1	1325 CEW deployments	-	4 (M26)	B
	UK, 2001–2002	2	1104 UOF entries	-	2,3,5	
	US, -	3	-	-	4	
	US, -	4	-	-	1,2,3,4(“pre-M26-weaponry”)	
	US, -	5	-	-	1,2,3,4(M26),5	
	US, -	6	-	-	4	
28	Lee et al., US -		-	-	4	B
40	Lin et al., US 2005–2007		1188 UOF incidents	80.3%	4	A
41	MacDonald et al., US 1998–2007		24,380 UOF incidents	87.7%	1,3,4	B
25	Maguire, US 2018–2020		2119 UOF incidents	92.5%	1,2,3,4,5	A
33	Petersen et al., US 2015–2019		2348 UOF incidents	90%	3,4,5,8	B
36	Sheppard et al., CA 2010–2018		17,155 UOF incidents 17,536 subjects 22,155 force utilizations	92.2%	1,2,3,4	A
30	Smith et al., US 2005–2006	1	467 UOF reports	-	1,2,3,4(X26),5	A
	2002–2006	2	762 UOF reports	90%	1,4,5	
31	Stevenson & Drummond-Smith, UK 2017		34,217 UOF	-	1,2,3,4(X26),5	B
42	Stroshine & Brandl, US 2014		662 UOF incidents	85.1%	1,3,4	A
37	Taylor & Woods, US 2000–2005		2234 suspect injuries 9131 suspect injuries	85.7%	4	B
29	Terril & Paoline III, US -		13,913 UOF cases	84%	1,2,3,4	A

1: Physical force, 2: Baton, 3: OC spray, 4: CEW (model listed in parenthesis when reported), 5: Canine, 6: Less-lethal (composite group, consisting of OC spray, pepperball launcher, CEW, rifles and shotguns using less lethal shells, riot shield), 7: Other physical methods (composite group, consisting of various forms of physical force, impact weapon such as a baton, canine, or the forcible stop of a fleeing vehicle), 8: Impact weapon (composite group, consisting of baton, flashlight, tear-gas, rubber bullets)

Injuries following exposure to UOF were largely based on data on UOF incidents from registers kept by law enforcement agencies (e.g., UOF forms). Additionally, two studies collected data on injuries as a result of UOF from national surveillance system databases (e.g., Centers for Disease Control and Prevention (CDC)) or data from a CEW manufacturer (e.g., Taser International) [26, 34, 35]. However, the definition of a UOF incident was not clear in every study; for example, whether an incident was characterized by a police intervention or by the number of force methods in a police intervention. A UOF incident might also include multiple officers or subjects, or multiple modalities. Only two articles presented sufficient data on UOF incidents (police encounters with a subject), subjects, and the total amount of force utilized (number of applied force modalities) [24, 36]. The number of UOF incidents reported in the included articles ranged from 467 to 34,217, resulting in 117,789 incidents (Table 2). Besides reporting on UOF incidents, data presentation was based on the number of CEW deployments, the number of patients treated at hospitals, or the number of observed injuries owing to police UOF (Table 2) [26, 34, 35, 37].

As data were based on law enforcement registries, injuries were often defined and registered by police personnel who were obliged to fill out UOF forms. However, two articles allowed physicians to review the data from police agencies together with medical records, classifying the injuries according to severity (mild, moderate, or severe) [24, 38]. Two articles reported using trained coders to read police reports and code content [35, 39]. Another approach was to create an injury severity index in categories of subjects voicing an injury, visible injuries as noted by police, transportation to hospital, subjects in need of medical attention, or subjects hospitalized [29, 33, 37, 40]. Two articles clearly stated that puncture wounds from probes of a CEW were not defined as an injury [41, 42], while this was not apparent in the other articles. Five articles reported on specific injuries/diagnoses following exposure to force modalities [26, 30, 35, 38, 39]. With reference to our quality assessment, 9 articles were deemed high quality (A), while the remaining 10 scored low quality (B) (Table 2).

The rate of subject injury following exposure to CEW ranged from 0 to 75.6% (Table 3a). The weighted mean injury rate across all articles reporting on subject injury following CEW was 24.5%. The rate was similar across the high-quality articles (26.8%) (Table 3b). Subject injury following OC spray ranged from 2.3 to 74.5% (Table 3a). The weighted mean of subject injury for all articles and high-quality articles exclusively were 20.2% and 6%, respectively (Table 3b). The rates of injury to subjects exposed to baton, canine, and physical force ranged from 3.8 to 80%, 4–100%, and 11–78%, respectively (Table 3a). The corresponding weighted mean injury rates for all and high-quality articles were 30.4% and 40.7% following the use of baton, 43.4% and 95.7% following the use of canines, and 59.4% and 31.4% following the use of physical force (Table 3b). In comparison to the weighted mean injury rate, the mean injury rate for the use of baton and canine across all articles were higher (44.8% and 62%, respectively). The rate of subject injury following the composite group *impact weapons* (batons, flashlights, tear gas, and rubber bullets) was 74% (Table 3a).

**Table 3a:**
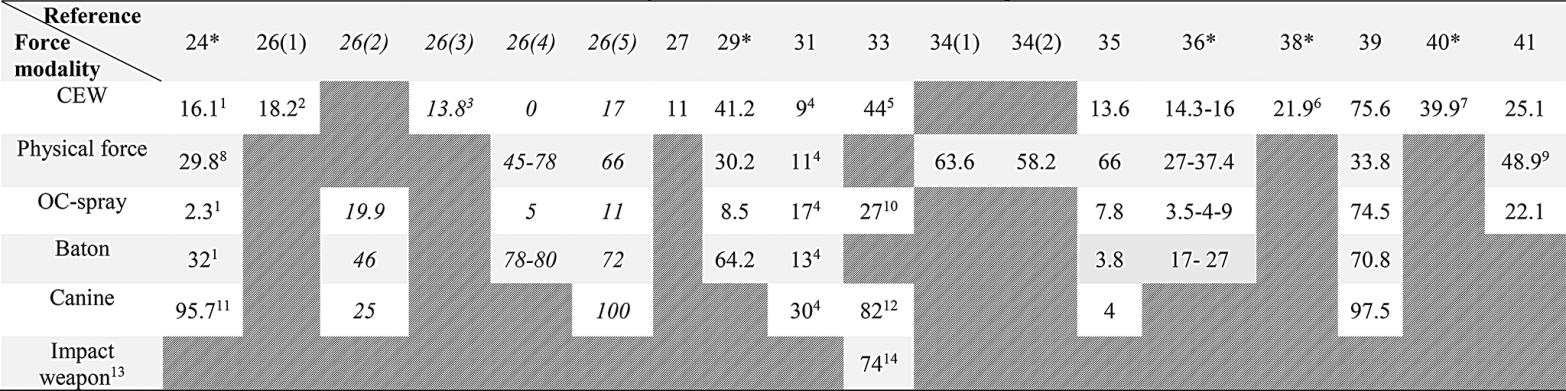
Subject injury rates following different use of force modalities as reported in the included studies

* High-quality article (quality A)

Number in parenthesis corresponds to source of the particular reference

Numbers in *cursive*: The reference is lacking number on incidents why these numbers are not included in the weighted mean rates (Table 3b)

^1^ Only mild injuries (minor contusions, lacerations, abrasion)

^2^ Pooled: Minor injuries (16.5%), moderate injuries (1.7%)

^3^ Pooled: Minor injuries (12.6%, moderate injuries (1.6%), severe injuries (0.6%)

^4^ Injuries from minor to significant, including injuries from the darts

^5^ Pooled data. Injury categories: Injured but not hospitalized (33%), injured and hospitalized or killed (11%)

^6^ Pooled data. Mild injuries (21.6%), moderate injuries (0.2%), severe injuries (0.1%)

^7^ Pooled data. Injury categories: Complained of (3.8%), visible (19.9%), transported to hospital (15.6%)

^8^ Pooled data. Mild injuries (29%), moderate injuries (0.7%), severe injuries (0.14%)

^9^ Including batons and flashlights

^10^ Pooled data. Injury categories: Injured but not hospitalized (25%), injured and hospitalized or killed (2%)

^11^ Pooled data. Mild injuries (85.1%), moderate injuries (10.6%)

^12^ Pooled data. Injury categories: Injured but not hospitalized (32%), injured and hospitalized or killed (50%)

^13^ Impact weapons: baton, flashlight, tear-gas, rubber bullets

^14^ Pooled data. Injury categories: Injured but not hospitalized (50%), injured and hospitalized or killed (24%)

**Table 3b:** Mean and weighted mean subject injury rates of all and quality A articles (standard deviation in parenthesis)

	Mean injury rates		Weighted mean injury rates	
	All articles	Quality A	All articles	Quality A
CEW	24.1 (SD: 18.9)	26.8 (SD: 12.8)	24.5 (SD: 19.3)	26.8 (SD: 12.7)
Physical force	46 (SD:19.4)	30.8 (SD: 14.6)	59.4 (SD: 12.4)	31.4 (SD: 1.2)
OC-spray	18.1 (SD: 20.4)	5 (SD: 3.2)	20.2 (SD: 18.9)	6 (SD: 2.2)
Baton	44.8 (SD: 28.2)	39.4 (SD: 22)	30.4 (SD: 24.3)	40.7 (SD: 20.7)
Canine	62 (SD: 40.8)	95.7 (SD: 0)	43.4 (SD: 31)	95.7 (SD: 0)

In terms of risk ratios for subject injury following exposure to less-lethal force modalities, three out of four articles indicated a decrease of subject injury following CEW by 65–87% compared to all other uses of force [30, 41, 42] (Table 4a). However, when compared specifically to physical force and OC spray, CEW increased the likelihood of subject injury [29]. In contrast, physical force was generally associated with an increase in risk of injuries compared to all other uses of less-lethal force, except for one study [25, 30, 39, 41]. The use of canines as a force modality increased the likelihood of subject injury regardless of reference category (all other use of force, CEW, and physical force) [24, 25, 30, 33]. In turn, OC spray reduced the risk of subject injury by 69–92% in comparison to all other types of force and 81% compared to CEW specifically [30, 33, 41, 42]. The composite group *less-lethal weapons* including OC spray, pepper ball launchers, CEW, rifles and shotguns using less lethal shells, and riot shields reduced subject injury by 51% in comparison to the composite group *other physical methods* including various forms of physical force, impact weapons such as batons, canines, and the forcible stop of a fleeing vehicle [32] (Table 4a).

**Table 4a:** Risk ratios of subject injury following different use of force modalities

Force modality		Force modality	Risk ratio	Reference
CEW	vs.	All other use of force	0.13 0.17 0.35 13.75	30(2) 42 41 25
CEW	vs.	Physical force	1.65	29
CEW	vs.	OC spray	3.04	29
CEW	vs.	Impact weapons^1^	0.4	29
Physical Force	vs.	All other use of force	0.23 1.56 2.47^2^ 2.54^2^ 4.68^3^ 31.81	39 41 30(1) 30(2) 30(2) 25
OC spray	vs.	All other use of force	0.08 0.31 0.31	42 41 30(1)
OC spray	vs.	CEW	0.19	33
Canine	vs.	All other use of force	20.54 41.37 1570	30(2) 30(1) 25
Canine	vs.	CEW	21.05 26.5	33 33
Canine	vs.	Physical force	26.1	24
Less-lethal impact munition^4^	vs.	All other use of force	9.45	25
Less-lethal^5^	vs.	Other physical method^6^	0.49	32

Number in parenthesis corresponds to source of the particular reference

^1^ Impact weapons: consisting of baton, bean bag, canine, and firearm

^2^ Hard hands (strikes and takedowns)

^3^ Soft hands (joint lock and pressure points)

^4^ Less-lethal impact munition: consisting of ARWEN, flex baton, pepperballs

^5^ Less-lethal: consisting of OC spray, pepperball launcher, CEW, rifles and shotguns using less lethal shells, riot shield

^6^ Other physical method: consisting of various forms of physical force, impact weapon such as a baton, canine, or the forcible stop of a fleeing vehicle

**Table 4b:** Risk ratios of subject injuries following implementation of CEW in agencies

Risk ratio	Reference
0.22^1^ 0.32–0.6^2^ 0.47–0.7^2^ 0.56^1^ 6.4^2,3^	37(1) 26(6) 41 37(2) 28

Number in parenthesis corresponds to source of the particular reference

^1^ Compared to non-CEW agencies

^2^ Compared to time period pre-CEW implementation

^3^ In-custody sudden death in the absence of lethal force, one year post-implementing CEW

Four articles reported on risk ratios of subject injury before and after implementation of CEW, alternatively in comparison with agencies not using CEW as a force modality (Table 4b). Three out of four studies reported a 30–78% reduction of subject injury after introduction of CEW [26, 37, 41]. However, after the implementation of CEW in several US custodies higher death rates among prisoners were observed [28].

Specific injuries following CEW were often mild such as superficial puncture wounds from probes or blunt trauma following falls (contusions, lacerations, superficial burn marks, fractures of finger and nasal bones). Moderate or severe injuries were rare and included head injuries such as temporoparietal intraparenchymal contusion and cerebellar epidural hematoma, or rhabdomyolysis [38]. Two subjects having been in an intensive struggle with the police collapsed unexpectedly 20 and 5 min, respectively, after exposure to CEW. Both subjects died. At autopsy one subject had dilated cardiomyopathy, and cocaine present in the blood, the other subject had antipsychotic drugs present in the blood. In both cases, no information on cause of death was given. However, the medical examiner determined that CEW was not a contributing factor [38]. Other injuries following CEW were internal injuries (not further specified) or teeth fractures [35, 39]. The specific injuries observed following physical force were bone fractures, traumatic brain injuries, and hemopneumothorax [24]. In addition, one cardiac arrest in association with physical force was reported; however, a causal association between death and UOF exposure was not established [24]. Finally, injuries associated with canines include bites to the extremities, bruises, and fractures [24, 26]. Such injuries were also seen following OC spray and the use of baton [26].

Regarding conflict of interest or funding, eight articles clearly stated that there were no competing interests [24, 25, 31, 34, 35, 38, 39, 42], four articles had a biography on authors in which there appeared to be no conflict of interest [29, 36, 37, 41]. In six articles there was no clear statement of competing interest, nor did the biography of authors allow for transparency [27, 28, 30, 32, 33, 40]. In one article one of the authors had undertaken a literature review paid for by a CEW manufacturer [26].

## Discussion

In summary, based on reporting on subject injury rates in real-life interventions in high-quality articles, CEW was associated with less injuries compared to baton, canine and physical force, but associated with more injuries compared to OC-spray. Considering the weighted mean injury rates of all included articles, physical force was associated with most injuries followed by canine and baton, whereafter CEW and OC spray followed. The result seems to be sensitive to the selection of articles included, as an example the weighted mean of subject injury following OC spray was 20.2% when all articles were included, focusing only on those articles rated as high quality (A) the rate was 6%. For CEW, five of 12 articles (5/12) were deemed high quality (Table 3a). A similar low proportion of high-quality articles were observed for physical force (3/8), OC spray (3/8), baton (3/6) and canine (1/5).

In analyses using risk ratios, the results for CEW were diverse; generally, the risk for subject injury were lowered compared to all other types of force but increased when compared specifically to physical force and OC spray. In turn, implementing CEW as a force modality seemed to result in a decrease in subject injury compared to non-CEW agencies. Physical force was generally associated with an increase in subject injury compared to all uses of force. However, the interpretation of risk ratio was not straightforward, as the reference categories were often not completely defined in the articles.

These findings could be representative, however are more likely indicative of the challenging task of comparing subject injuries following UOF across several sources in different contexts with varying approaches on registering injuries and tactical recommendations. Hence, the results of this inquiry should be interpreted with care for methodological reasons. The included studies were heterogeneous in design. Although 9 articles were deemed high quality, including aspects of transparency on registration of injuries, only a few studies allowed for further categorization such as injury severity. Thus, many studies had significant weaknesses, such as not ascertaining that all cases with injuries following exposure to UOF were identified, not using a transparent approach to the classification of injuries and not ascertaining which modality of force specifically caused the observed injury when multiple modalities were used. For instance, if the use of multiple weapons against the same individual led to injury, the injury would be recorded against each UOF modality, regardless of which weapon had caused the injury [31]. In only two articles, it was evident that puncture wounds following CEW probes were not defined as injuries [41, 42]. In addition, transparency was often lacking regarding usage of CEW as a deterrent (e.g., if displaying or drawing without discharging the probes counted as one incident or not).

A crucial factor in evaluating the severity of injuries is the preexisting health of the subjects exposed to UOF. Using the current study design, it is difficult to separate the importance of the context from the direct effects of UOF regarding the risk of injury. It is reasonable to assume that in situations in which the subject is agitated and aggressive, possibly armed or attacking police officers or other subjects, the risk of injury increases [32]. Such a setting also highlights the importance of categorizing subject injuries as either following a specific modality of force or to the situation itself, including falls, other accidents, or injuries inflicted by a third party aggressor.

Information on the legislative framework and local tactical police guidelines for consideration of which modality to be used in a specific situation did not appear clearly in any of the included articles. The tactical approach of police agencies could be hypothesized to be important for the occurrence of injuries and the level of aggression in a situation. This aspect is difficult to capture using the study designs applied in the current literature. A suggestion for future research is to report on the national legal framework and tactical guidelines for local practices for police UOF.

A previous review on the harm-reducing effects of less-lethal UOF modalities in a real-life setting for both subjects and officers identified 12 articles of interest [43]. The review reached the overall conclusion that CEW did not have a significant effect on citizen injury. Importantly, the included studies on CEW policy changes suggested that lowering CEW on the UOF continuum was associated with a reduction in overall harm [44, 45]. However, this might reflect that a more liberal use of CEW, replacing physical force in low-risk cases, equals lower subject injury. On the contrary, using CEW only in high-risk cases (e.g., encountering an aggressive subject under the influence of drugs and in possession of weapons) would likely be associated with an increase in subject injury.

We suggest that studies reporting risks following the use of different modalities of force in operative police work be systematic, so all exposed cases in a defined area are registered, all cases exposed to UOF are medically examined, and each injury is coupled with the most likely causative modality of force. Since no consensus seems to exist on how to define and count an injury following the use of a less-lethal force (e.g., puncture wounds inflicted by CEW probes), we suggest establishing injury protocols for each modality of force to achieve an international standard for systematic UOF research. We also suggest that the medical community should agree on an injury scale to be applied to categorizing injuries following exposure to the different modalities of force applied by law enforcement to generate comparable results across studies. The injury scale needs to be adapted to suit each modality of force (e.g., a consensus on whether or not insignificant skin lesions following CEW probes should be registered as lesions). In addition, the preexisting health of individuals exposed to UOF by law enforcement should be reported together with information on the results of toxicology screenings.

UOF is a powerful tool in upholding law and order; however, if abused, it is a potential source of harm and suffering. Establishing an evidence base for the rate and type of injuries following UOF allows for more proper guidelines for law enforcement, as well as opportunities for surveillance of use and international comparisons.

### Strengths and limitations

This review is based on a comprehensive literature search applying different search terms and synonyms for less-lethal UOF modalities, injury, and UOF in three databases. The application of a modified quality assessment tool increased the strength of the results section, pointing out the heterogeneity of the results when comparing the weighted mean rates of subject injury in all articles versus high-quality articles. Thus, this might allow for a more distinct interpretation of subject injury rates.

Most of the articles concerned the use of CEW. This might reflect that our extensive search string on CEW “favored” CEW in comparison to the other less-lethal weapons. A more thorough search string and adding more synonyms for other less-lethal modalities could have increased the impact of those modalities. As we managed to generate a rate and risk ratio for subject injury, we did not acknowledge the context of how these injuries were sustained. Independent variables, such as subjects under drug/alcohol influence, subject resistance, time to restraint, rank and assignment of the officer involved, and the underlying reason for the encounter, could all have modeled the outcome of the subject injury. The year of data spanned from 1998 to 2020 and only a few articles reported on the model of CEW used by the police. This is a limitation since new generations of CEW with different electrophysiological properties are readily available for agencies [46, 47]. Only a few articles reported specifically on serious subject injuries. Following this, the frequency of serious injuries could not be determined separately. Most studies reported data on UOF incidents, while other studies reported UOF reports, UOF entries, or UOF cases. For the most part, it was uncertain how these terms were defined. This is a limitation of our study, as we applied the terms synonymously. The body of the included literature consisted of 19 articles with heterogeneous study designs. A meta-analysis with the weighted mean injury rates was performed, yet it is uncertain whether a meta-analysis was appropriate with the inclusion of several heterogeneously designed studies. Thus, the evidence for the conclusions of this review is weak, and there is a need for more extensive research on injuries caused by UOF by law enforcement in real-life settings using a systematic and transparent approach.

## Conclusion

The body of literature comparing injury rates between different UOF modalities in operative police work is sparse. In this study, we identified 19 articles, all of which were heterogeneous in design, thus limiting the conclusions drawn. All less-lethal modalities are associated with the risk of harm. OC spray proved to have the lowest risk of subject injury. The outcome of subject injury following CEW was ambiguous; however, there is an indication that the risk of injuries following CEW is lowered in comparison to baton and canine. However, the observed associations might reflect the context of the police intervention, such as applied police tactics, subject agitation, and level of aggression in the situation. Therefore, further research using a systematic approach is imperative to report on injuries, the context of the situation, legislative framing, and local recommendations on tactical UOF.

## Key points

1. The evidence base for classifying the risk of injury according to the modality of force in real-life settings is weak.
2. There is an indication that the risk for injuries following exposure to conducted electrical weapons in a real-life setting is lower than the use of baton and canines. OC spray appeared to have the lowest rate of subject injury.
3. Future research on injuries following the use of force in real-life settings should include systematic registration of lesions, context, and preexisting health conditions.
4. The legislative framework and the local procedures that guide the use of force in operative police work is important in research on the use of force.

## Electronic supplementary material

Below is the link to the electronic supplementary material.

Supplementary Material 1
