# Supplementary material for: Injury rates following conducted electrical weapons and other less-lethal force modalities in real-life police settings: a comparative literature review
Source: Forensic Sci Med Pathol. 2025 May 15;21(3):1396–406. doi: 10.1007/s12024-025-01020-9 (PMC12491360; doi:10.1007/s12024-025-01020-9)
Supplement: Supplementary file 1 — Supplementary Material 1 [file 12024_2025_1020_MOESM1_ESM.docx]

**Supplement 1: Search string**

PubMed

Date of search: 8^th^ of January 2025

| **Topic** | **Search terms** | **Results** |
| --- | --- | --- |
| Conducted Electrical Weapon | ((((((((((((((((((((((((((((("CEDs") OR ("CEWs")) OR ("conducted electrical weapon*")) OR ("conducted energy device*")) OR ("conducted energy weapon*")) OR ("conductive electrostimulation*")) OR ("conductive energy device*")) OR ("conductive energy weapon*")) OR ("electric shock device*")) OR ("electric shock exposure*")) OR ("electric shock weapon*")) OR ("electrical stun gun*")) OR ("electrical weapon*")) OR ("electronic control device*")) OR ("electronic weapon*")) OR ("electroshock device*")) OR ("electroshock weapon*")) OR ("energy weapon*")) OR ("Human electro muscular incapacitation*")) OR ("impact weapon*")) OR ("incapacitation device*")) OR ("less lethal force*")) OR ("less lethal kinetic*")) OR ("less lethal technolog*")) OR ("less lethal weapon*")) OR ("neuromuscular incapacit*")) OR ("non lethal exposure*")) OR ("stun gun*")) OR ("Taser*")) AND (("Conducted Energy Weapon Injuries"[Mesh]) OR (("Wounds and Injuries"[Mesh]) OR (("adverse health effects") OR (injur*)))) Filters: English, German, from 2000 - 2025 | 21 |
|  | ((((((((((((((((((((((((((((("CEDs") OR ("CEWs")) OR ("conducted electrical weapon*")) OR ("conducted energy device*")) OR ("conducted energy weapon*")) OR ("conductive electrostimulation*")) OR ("conductive energy device*")) OR ("conductive energy weapon*")) OR ("electric shock device*")) OR ("electric shock exposure*")) OR ("electric shock weapon*")) OR ("electrical stun gun*")) OR ("electrical weapon*")) OR ("electronic control device*")) OR ("electronic weapon*")) OR ("electroshock device*")) OR ("electroshock weapon*")) OR ("energy weapon*")) OR ("Human electro muscular incapacitation*")) OR ("impact weapon*")) OR ("incapacitation device*")) OR ("less lethal force*")) OR ("less lethal kinetic*")) OR ("less lethal technolog*")) OR ("less lethal weapon*")) OR ("neuromuscular incapacit*")) OR ("non lethal exposure*")) OR ("stun gun*")) OR ("Taser*")) AND (("Conducted Energy Weapon Injuries"[Mesh]) OR (("Wounds and Injuries"[Mesh]) OR (("adverse health effects") OR (injur*)))) Filters: English, German, from 2000 – 2025 | 346 |
| Physical force | (("physical force*") OR ("empty hand")) AND (("Wounds and Injuries"[Mesh]) OR (("adverse health effects") OR (injur*))) Filters: English, German, from 2000 - 2025 | 162 |
|  | (("physical force*") OR ("empty hand")) AND (("police use of force") OR ("use of force")) Filters: English, German, from 2000 - 2025 | 14 |
| Baton | (baton* OR truncheon*) AND (("use of force") OR ("police use of force")) Filters: English, German, from 2000 - 2025 | 3 |
|  | (baton* OR truncheon*) AND (("Wounds and Injuries"[Mesh]) OR (("adverse health effects") OR (injur*))) Filters: English, German, from 2000 - 2025 | 685 |
| Canine | ((K9) OR (canine*) OR ("police dog*") AND ((police*) OR (law enforcement*))) AND (("police use of force") OR ("use of force")) Filters: English, German, from 2000 - 2025 | 8 |
|  | ((K9) OR (canine*) OR ("police dog*") AND ((police*) OR (law enforcement*))) AND (("Wounds and Injuries"[Mesh]) OR (("adverse health effects") OR (injur*))) Filters: English, German, from 2000 - 2025 | 53 |
| OC-spray | ((("oleoresins" [Supplementary Concept]) OR ("OC spray*")) OR ("pepper spray*")) AND (("police use of force") OR ("use of force")) Filters: English, German, from 2000 - 2025 | 1 |
|  | (((("oleoresins" [Supplementary Concept]) OR ("OC spray*")) OR ("pepper spray*"))) AND (("Wounds and Injuries"[Mesh]) OR (("adverse health effects") OR (injur*))) Filters: English, German, from 2000 - 2025 | 37 |
| Less-lethal weapon | Search: (("police use of force") OR ("use of force")) AND (("less-lethal weapon*") OR ("non-lethal weapon*"))  Filters: English, German, from 2000 - 2025 | 8 |
|  | (("less-lethal weapon*") OR ("non-lethal weapon*")) AND (("Wounds and Injuries"[Mesh]) OR (("adverse health effects") OR (injur*)))  Filters: English, German, from 2000 - 2025 | 59 |

Embase

Date of search: 8^th^ of January 2025

| **Topic** | **Search terms** | **Results** |
| --- | --- | --- |
| 1 - Police use of force | ("police use of force" or "use of force").mp. [mp=title, abstract, heading word, drug trade name, original title, device manufacturer, drug manufacturer, device trade name, keyword heading word, floating subheading word, candidate term word] | 1193 |
| 2 - Injuries | ("Wounds and Injuries" or "adverse health effects" or injur*).mp. [mp=title, abstract, heading word, drug trade name, original title, device manufacturer, drug manufacturer, device trade name, keyword heading word, floating subheading word, candidate term word] | 2140193 |
| Conducted Electrical Weapon | ("CEDs" or "CEWs" or "conducted electrical weapon*" or "conducted energy device*" or "conducted energy weapon*" or "conductive electrostimulation*" or "conductive energy device*" or "conductive energy weapon*" or "electric shock device*" or "electric shock exposure*" or "electric shock weapon*" or "electrical stun gun*" or "electrical weapon*" or "electronic control device*" or "electronic weapon*" or "electroshock device*" or "electroshock weapon*" or "energy weapon*" or "Human electro muscular incapacitation*" or "impact weapon*" or "incapacitation device*" or "less lethal force*" or "less lethal kinetic*" or "less lethal technolog*" or "less lethal weapon*" or "neuromuscular incapacit*" or "non lethal exposure*" or "stun gun*" or "Taser*").mp. [mp=title, abstract, heading word, drug trade name, original title, device manufacturer, drug manufacturer, device trade name, keyword heading word, floating subheading word, candidate term word] AND 1  Limit ((english or german) and yr="2000 - 2025") | 37 |
|  | ("CEDs" or "CEWs" or "conducted electrical weapon*" or "conducted energy device*" or "conducted energy weapon*" or "conductive electrostimulation*" or "conductive energy device*" or "conductive energy weapon*" or "electric shock device*" or "electric shock exposure*" or "electric shock weapon*" or "electrical stun gun*" or "electrical weapon*" or "electronic control device*" or "electronic weapon*" or "electroshock device*" or "electroshock weapon*" or "energy weapon*" or "Human electro muscular incapacitation*" or "impact weapon*" or "incapacitation device*" or "less lethal force*" or "less lethal kinetic*" or "less lethal technolog*" or "less lethal weapon*" or "neuromuscular incapacit*" or "non lethal exposure*" or "stun gun*" or "Taser*").mp. [mp=title, abstract, heading word, drug trade name, original title, device manufacturer, drug manufacturer, device trade name, keyword heading word, floating subheading word, candidate term word] AND 2  Limit ((english or german) and yr="2000 - 2025") | 342 |
| Physical force | ("physical force*" or "empty hand").mp. [mp=title, abstract, heading word, drug trade name, original title, device manufacturer, drug manufacturer, device trade name, keyword heading word, floating subheading word, candidate term word]  AND 1  Limit ((english or german) and yr="2000 - 2025") | 34 |
|  | ("physical force*" or "empty hand").mp. [mp=title, abstract, heading word, drug trade name, original title, device manufacturer, drug manufacturer, device trade name, keyword heading word, floating subheading word, candidate term word]  AND 2  Limit ((english or german) and yr="2000 - 2025") | 245 |
| Baton | (baton* or truncheon).mp. [mp=title, abstract, heading word, drug trade name, original title, device manufacturer, drug manufacturer, device trade name, keyword heading word, floating subheading word, candidate term word]  AND 1  Limit ((english or german) and yr="2000 - 2025") | 5 |
|  | (baton* or truncheon).mp. [mp=title, abstract, heading word, drug trade name, original title, device manufacturer, drug manufacturer, device trade name, keyword heading word, floating subheading word, candidate term word]  AND 2  Limit ((english or german) and yr="2000 - 2025") | 66 |
| Canine | ((K9 or canine* or "police dog*") and (police* or law enforcement*)).mp. [mp=title, abstract, heading word, drug trade name, original title, device manufacturer, drug manufacturer, device trade name, keyword heading word, floating subheading word, candidate term word]  AND 1  Limit ((english or german) and yr="2000 - 2025") | 9 |
|  | ((K9 or canine* or "police dog*") and (police* or law enforcement*)).mp. [mp=title, abstract, heading word, drug trade name, original title, device manufacturer, drug manufacturer, device trade name, keyword heading word, floating subheading word, candidate term word]  AND 2  Limit ((english or german) and yr="2000 - 2025") | 47 |
| OC-spray | ("oleoresins" or "OC spray*" or "pepper spray*").mp. [mp=title, abstract, heading word, drug trade name, original title, device manufacturer, drug manufacturer, device trade name, keyword heading word, floating subheading word, candidate term word]  AND 1  Limit ((english or german) and yr="2000 - 2025") | 5 |
|  | ("oleoresins" or "OC spray*" or "pepper spray*").mp. [mp=title, abstract, heading word, drug trade name, original title, device manufacturer, drug manufacturer, device trade name, keyword heading word, floating subheading word, candidate term word]  AND 2  Limit ((english or german) and yr="2000 - 2025") | 46 |
| Less-lethal weapon | ("less-lethal weapon*" or "non-lethal weapon*").mp. [mp=title, abstract, heading word, drug trade name, original title, device manufacturer, drug manufacturer, device trade name, keyword heading word, floating subheading word, candidate term word]  AND 1  Limit ((english or german) and yr="2000 - 2025") | 8 |
|  | ("less-lethal weapon*" or "non-lethal weapon*").mp. [mp=title, abstract, heading word, drug trade name, original title, device manufacturer, drug manufacturer, device trade name, keyword heading word, floating subheading word, candidate term word]  AND 2  Limit ((english or german) and yr="2000 - 2025") | 64 |

Web of Science

Date of search: 8^th^ of January 2025

| **Topic** | **Search terms** | **Results** |
| --- | --- | --- |
| Conducted Electrical Weapon | (((((((((((((((((((((((((((("CEDs") OR ("CEWs")) OR ("conducted electrical weapon*")) OR ("conducted energy device*")) OR ("conducted energy weapon*")) OR ("conductive electrostimulation*")) OR ("conductive energy device*")) OR ("conductive energy weapon*")) OR ("electric shock device*")) OR ("electric shock exposure*")) OR ("electric shock weapon*")) OR ("electrical stun gun*")) OR ("electrical weapon*")) OR ("electronic control device*")) OR ("electronic weapon*")) OR ("electroshock device*")) OR ("electroshock weapon*")) OR ("energy weapon*")) OR ("Human electro muscular incapacitation*")) OR ("impact weapon*")) OR ("incapacitation device*")) OR ("less lethal force*")) OR ("less lethal kinetic*")) OR ("less lethal technolog*")) OR ("less lethal weapon*")) OR ("neuromuscular incapacit*")) OR ("non lethal exposure*")) OR ("stun gun*")) OR ("Taser*") AND **("police use of force") OR ("use of force")**  Filter: 2000 - 2025, Language: English, German | 95 |
|  | (((((((((((((((((((((((((((("CEDs") OR ("CEWs")) OR ("conducted electrical weapon*")) OR ("conducted energy device*")) OR ("conducted energy weapon*")) OR ("conductive electrostimulation*")) OR ("conductive energy device*")) OR ("conductive energy weapon*")) OR ("electric shock device*")) OR ("electric shock exposure*")) OR ("electric shock weapon*")) OR ("electrical stun gun*")) OR ("electrical weapon*")) OR ("electronic control device*")) OR ("electronic weapon*")) OR ("electroshock device*")) OR ("electroshock weapon*")) OR ("energy weapon*")) OR ("Human electro muscular incapacitation*")) OR ("impact weapon*")) OR ("incapacitation device*")) OR ("less lethal force*")) OR ("less lethal kinetic*")) OR ("less lethal technolog*")) OR ("less lethal weapon*")) OR ("neuromuscular incapacit*")) OR ("non lethal exposure*")) OR ("stun gun*")) OR ("Taser*")  AND ("Wounds and Injuries” OR (("adverse health effects") OR (injur*))  Filter: 2000 - 2025, Language: English, German | 300 |
| Physical force | (("physical force*") OR ("empty hand") AND (("police use of force") OR ("use of force"))  Filter: 2000 - 2025, Language: English, German | 56 |
|  | (("physical force*") OR ("empty hand") AND (("Wounds and Injuries” OR (("adverse health effects") OR (injur*)))  Filter: 2000 - 2025, Language: English, German | 198 |
| Baton | (baton*) OR (truncheon) AND (police OR law enforcement) AND (("Wounds and Injuries” OR (("adverse health effects") OR (injur*)))  Filter: 2000 - 2025, Language: English, German | 22 |
|  | (baton*) OR (truncheon) AND (police OR law enforcement) AND (("police use of force") OR ("use of force"))  Filter: 2000 - 2025, Language: English, German | 13 |
| Canine | ((K9) OR (canine*) OR ("police dog*")) AND ((police*) OR (law enforcement*)) AND (("police use of force") OR ("use of force"))  Filter: 2000 - 2025, Language: English, German | 10 |
|  | ((K9) OR (canine*) OR ("police dog*")) AND ((police*) OR (law enforcement*)) AND (("Wounds and Injuries"OR (("adverse health effects") OR (injur*)))  Filter: 2000 - 2025, Language: English, German | 57 |
| OC-spray | ((("oleoresins" [Supplementary Concept]) OR ("OC spray*")) OR ("pepper spray*")) AND (("police use of force") OR ("use of force"))  Filter: 2000 - 2025, Language: English, German | 22 |
|  | ((("oleoresins" [Supplementary Concept]) OR ("OC spray*")) OR ("pepper spray*")) AND (("Wounds and Injuries” OR (("adverse health effects") OR (injur*)))  Filter: 2000 - 2025, Language: English, German | 48 |
| Less-lethal weapon | ("less-lethal weapon*") OR ("non-lethal weapon*") AND (("police use of force") OR ("use of force"))  Filter: 2000 - 2025, Language: English, German | 15 |
|  | ("less-lethal weapon*") OR ("non-lethal weapon*") AND (("Wounds and Injuries"OR (("adverse health effects") OR (injur*)))  Filter: 2000 – 2025, Language: English, German | 73 |
